# SOX4 Mediates TGF-β-Induced Expression of Mesenchymal Markers during Mammary Cell Epithelial to Mesenchymal Transition

**DOI:** 10.1371/journal.pone.0053238

**Published:** 2013-01-03

**Authors:** Stephin J. Vervoort, Ana Rita Lourenço, Ruben van Boxtel, Paul J. Coffer

**Affiliations:** 1 Department of Cell Biology, University Medical Centre, Utrecht, The Netherlands; 2 Division of Pediatrics, Wilhelmina Children’s Hospital, University Medical Centre, Utrecht, The Netherlands; Huntsman Cancer Institute, University of Utah, United States of America

## Abstract

The epithelial to mensenchymal transition program regulates various aspects of embryonic development and tissue homeostasis, but aberrant activation of this pathway in cancer contributes to tumor progression and metastasis. TGF-β potently induces an epithelial to mensenchymal transition in cancers of epithelial origin by inducing transcriptional changes mediated by several key transcription factors. Here, we identify the developmental transcription factor *SOX4* as a transcriptional target of TGF-β in immortalized human mammary epithelial cells. SOX4 expression and activity are rapidly induced in the early stages of the TGF-β-induced epithelial to mensenchymal transition. We demonstrate that conditional activation of Sox4 is sufficient to induce the expression of N-cadherin and additional mesenchymal markers including vimentin and fibronectin, but fails to induce complete EMT as no changes are observed in the expression of E-cadherin and β-catenin. Moreover, shRNA-mediated knockdown of SOX4 significantly delays TGF-β-induced mRNA and protein expression of mesenchymal markers. Taken together, these data suggest that TGF-β-mediated increased expression of SOX4 is required for the induction of a mesenchymal phenotype during EMT in human mammary epithelial cells.

## Introduction

The epithelial to mesenchymal transition (EMT) program is a reversible process important during embryonic development and tissue homeostasis by controlling the formation of the body plan and tissue and organ differentiation [1]. Deregulation of EMT through incorrect or excessive activation can also result in adverse effects by inducing fibrosis and cancer progression [1]. Induction of EMT evokes a change from a polarized epithelial phenotype, in which cells are adherent to the basement membrane and express classical epithelial makers including E-cadherin and ZO-1, to a mesenchymal state in which cell-cell contact is lost and mesenchymal makers are expressed such as N-cadherin and Vimentin [2], [3]. TGF-β is a potent inducer of EMT in a wide variety of human cancers of epithelial origin. The EMT induced mesenchymal phenotype in epithelial cancer types is associated with increased migratory and invasive properties, basement membrane degradation, resistance to apoptosis and cancer stem cell characteristics, which ultimately results in increased metastasis, therapy resistance and poor-prognosis in cancer patients [2], [3], [4]. TGF-β-induced EMT is mediated by both the canonical Smad2/3 dependent pathway and the non-canonical Smad2/3-independent pathway which includes the MAPK and PI-3K/PKB signaling routes [5]. The phenotypic changes observed during TGF-β-induced EMT are exerted through alterations in the expression of a variety of transcriptional regulators, including Snail, Slug, Twist, Goosecoid, zinc-finger E-box binding homeobox 1 (ZEB1) and FOXC2 [4]. Most of these transcription factors are transcriptional repressors involved in the direct or indirect down-regulation of E-cadherin expression and a reduction in the epithelial phenotype. In contrast, the TGF-β-mediated induction of a mesenchymal phenotype during EMT appears to be controlled by transcriptional activators. For example, TGF-β-mediated induction of the transcription factor FOXC2 has been shown to be required for the increased expression of mesenchymal markers such as N-cadherin, vimentin and fibronectin [6], [7]. However, ectopic expression of FOXC2 in epithelial cells is insufficient to induce a full EMT phenotype resulting in increased expression of mesenchymal markers, but lacking complete repression of E-cadherin and other epithelial markers [7]. In this study we investigated the potential role of additional transcriptional activators in the context of TGF-β-induced EMT in breast cancer. Here, we identify SOX4 as a transcriptional activator of which both the expression and transcriptional activity are induced by TGF-β in human mammary epithelial cells (HMECs) during EMT. Conditional activation of SOX4 in non-transformed immortalized HMEC expressing hTERT and SV40 large T and small t antigens (HMLE) was sufficient to drive the expression mesenchymal markers, such as N-cadherin, without affecting expression of the epithelial markers E-cadherin and β-Catenin. Finally, we demonstrate that SOX4 expression is necessary for TGF-β-mediated induction of N-cadherin during EMT. Taken together, these data identify SOX4 as a novel transcriptional activator involved in the transcriptional response regulating mesenchymal gene expression during TGF-β-induced EMT.

## Materials and Methods

### Cell Culture

Non-transformed Human mammary epithelial cells (classified as HMLE hTERT and kindly provided by Dr. Robert Weinberg) were cultured in MEGM medium (Lonza, Basel, Switzerland): F12 media (Invitrogen, Oregon, USA) (1∶1) supplemented with insulin (Lonza), EGF (Lonza), hydrocortisone (Lonza), penicillin-streptomycin (Invitrogen, Oregon, USA) (Weinberg et al, 2008). Mesenchymal-like phenotype cell cultures were obtained by supplementing the normal culture medium with 2.5 ng/ml of TGF-β1 (Sigma-Aldrich-Aldrich, Missouri, USA). HEK293T cells (derived from human embryonic kidney) were maintained in DNEM (Invitrogen) supplemented with 8% heat-inactivated FBS and penicillin-streptomycin (Invitrogen).

### Generation of a Sox4 Cell Lines

To generate a conditionally regulated Sox4 (ER:Sox4), the sequence of the mouse Sox4 gene was fused in frame with to the hormone-binding domain of the human estrogen receptor (ER). The ER:Sox4 or ER construct was subcloned into the polylinker region of the pBABE vector which contains an internal ribosomal entry site followed by the gene encoding for puromycin resistance. pBABE-puro retrovirus was produced by stable transfection of the retroviral packaging cell line, Phoenix-ampho, by calcium phosphate coprecipitation. Viral supernatants were collected, filtered through a 0.2-µm filter and 4 µg/µL of polybrene was added. HMLE cells were transduced overnight. Transduction was performed by adding 0.5 mL of viral supernatant to 0.5 mL of medium containing 0.5×10^6^ cells. During experiments, polyclonal HMLE ER and ER:Sox4 cell lines were maintained in MEGM (Lonza, Basel, Switzerland): F12 media (Invitrogen, Oregon, USA) (1∶1) supplemented with insulin (Lonza), EGF (Lonza), hydrocortisone (Lonza), penicillin-streptomycin (Invitrogen, Oregon, USA) (Weinberg et al, 2008) and stimulated with 100 nM of 4-hydroxy tamoxifen [(4-OHT), Sigma-Aldrich, Missouri, USA].

### shRNA Viral Transduction of HMLE Cells

A lentiviral construct was used expressing shRNA control [(SHC002); Sigma-Aldrich, Missouri, USA] or shRNA targeting Sox4 (TRCN0000018214, Sigma) and an internal ribosomal entry site followed by the gene encoding for puromycin resistance in the pLKO.1 vector (SHC001, Sigma). pLKO.1-puro lentivirus was produced by stable transfection of the retroviral packaging cell line, Phoenix-ampho, by calcium phosphate coprecipitation. Viral supernatants were collected, filtered through a 0.2-µm filter and 4 µg/µL of polybrene was added. HMLE cells were transduced overnight. Transduction was performed by adding 0.5 mL of viral supernatant to 0.5 mL of medium containing 0.5×10^6^ cells. During experiments, polyclonal shRNA control (Scr) and shRNA SOX4 cell lines were maintained in MEGM (Lonza, Basel, Switzerland): F12 media (Invitrogen, Oregon, USA) (1∶1) supplemented with insulin (Lonza), EGF (Lonza), hydrocortisone (Lonza), penicillin-streptomycin (Invitrogen, Oregon, USA) (Weinberg et al, 2008) and stimulated with 2.5 ng/ml of TGF-β1 (Sigma-Aldrich, Missouri, USA).

### Chromatin Immuno-precipitation (ChIP)

ChIP was performed as previously described [8]. MDA-MB-231 cells were crosslinked with 2 mM disuccinimidyl glutarate (Thermo Fisher Scientific) and 1% formaldehyde, cells were lysed in pre-immunoprecipitation buffer (10 mM Tris, 10 mM NaCl, 3 mM MgCl_2_ and 1 mM CaCl_2_). Chromatin was prepared and ChIP was performed according to the Millipore online protocol using 5 µg of antibodies against SOX4 or rabbit IgG as a control. The primers used for analysis are listed in Table 1.

**Table 1:** qRT-PCR primers.

Gene	Forward primer	Reverse primer
***CDH2*** ** −3900**	5′ tgggatgaaagggagattttt 3′	5′ aaaagcatatgaaaactgagagca 3′
***CDH2*** ** −2600**	5′ gatcacctggtcagccaaa 3′	5′ gcacaaagtctccaacagca 3′
***CDH2*** ** −1000**	5′ ggcagacacagcaaactaagg 3′	5′ gtgcgagctccagagagg 3′
***CDH2*** **+25000**	5′ aaagccatcctaggcagtca 3′	5′ atcctgccttgcttcttgg 3′
***CDH2*** **+29600**	5′ cattccacttggcataaagc 3′	5′ tgattaaccctttgccctct 3′
***Β2-Microglobulin***	5′ atgagtatgcctgccgtgtg 3′	5′ ggcatcttcaaacctccatg 3′
***CTNNB1***	5′ gaaggtgtggcgacatatgca 3′	5′ atccaaggggttctccctgggc 3′
***CDH1***	5′ caccacgtacaagggtcaggtgc 3′	5′ cagcctcccacgctggggtat 3′
***FN1***	5′ tggcaccccacgctcagataca 3′	5′ ctcgccaggcaggttgacgg 3′
***CDH2***	5′ agtcaccgtggtcaaaccaatcga 3′	5′ tgcagttgactgaggcgggtg 3′
***SOX4***	5′ ggcctcgagctgggaatcgc 3′	5′ gcccactcggggtcttgcac 3′
***VIM***	5′ accaacgacaaagcccgcgt 3′	5′ cagagacgcattgtcaacatcctgt 3′

### Quantification of RNA Expression

mRNA was extracted from HMLE cell lines using the Rneasy Isolation Kit (Qiagen, Copenhagen Denmark). According to the manufactures protocol for single-stranded cDNA synthesis, 500 ng of total RNA was reverse transcribed using iScript cDNA synthesis kit (BIO-Rad, Hercules, CA). cDNA samples were amplified using SYBR green supermix (BIO-Rad), in a MyiQ single-color real time PCR detection system (BIO-Rad) according to the manufacturés protocol. To quantify the data, the comparative Ct method was used. Relative quantity was defined as 2^− ΔΔCt^ and *β2-Microglobulin* was used as reference gene. The sequence of the primers are listed in Table 1.

### Western Blotting

Cells were washed with PBS and lysed in Laemmli buffer [0.12 mol/L Tris-HCL (pH 6.8), 4% SDS and 20% glycerol]. Protein concentration was determined using Lowry protein assay. Equal amounts of sample (30 µg) were analyzed by Sodium dodecyl sulfate polyacrylamide gel electrophoresis (SDS-PAGE) and electrophoretically transferred to polyvinylidene difluoride membrane (Milipore, Bedford, MA). The membranes were blocked with 5% milk protein in TBST (0.3% Tween, 10 mM Tris pH8 and 150 mM NaCl in H_2_O) and probed with antibodies as indicated in Table 2. Immunocomplexes were detected using ECL and exposure to Kodak XB films (Rochester, NY).

**Table 2:** Antibodies conditions for western blot analysis.

Antibody name	Supplier	Product number	Dilution
Anti-E-cadherin	BD transduction	610182	1∶3000
Anti-N-cadherin	BD transduction	610921	1∶1000
Anti-Sox4	Diagenode	CS-129-100	1∶3000
Anti-ERα	Santa Cruz Biotechnology	SC 542	1∶1000
Anti-tubulin	Sigma-Aldrich	T5168	1∶50000

### Confocal Microscopy

Cells were cultured on poly-L-lysine-coated microscope glasses (Sigma-Aldrich, Missouri, USA), Coverslips were washed with PBS before fixation using PBS containing 3% paraformaldehyde (Merck, Nottingham, United Kingdom) for 30 minutes at room temperature. Cells were preincubated with 10% normal bovine serum (Sigma) and 0.5% saponin (Sigma) for 15 minutes. Next, cells were incubated overnight with mouse anti-E-cadherin directed conjugated with Alexa Fluor 647 (BD Transduction; 1∶10) and mouse anti-N-cadherin (BD Transduction; 1∶100) antibodies in PBS containing 10% normal bovine serum and 0.5% saponin. Cells were washed three times with PBST (0.05% Tween), and mounted in Mowiol 4–88 (Sanofi-Aventis, Paris, France) containing DAPI. Confocal images were acquired using a Zeiss LSM 710 fluorescence microscope (Oberkochen, Germany).

### Biotinylated Oligonucleotide Pull Down Assay

HEK293T cells were transiently transfected with pcDNA3 or Flag-tagged Sox4, grown in 10 cm plates to ∼90% confluence and washed with PBS. Cells were lysed in 025 mM HEPES, 5 mM KCl, 0.5 mM MgCl_2,_ 1 mM DTT, 1% Halt Protease Inhibitor Cocktail (Thermo Scientific, Rockford, USA) and 2% Nonidet P-40 (US Biological, Massachusetts, USA). Nuclear fraction was extracted in 25 mM HEPES, 10% sucrose, 350 mM NaCl, 1 mM DTT and 1% Halt Protease Inhibitor Cocktail (Thermo Scientific). The mixture was vigorously shaken at 4°C for 1 hour and centrifuged at 4°C for 10 min at 25000 rcf. The supernatant (nuclear extract) was freshly used.

DNA-protein interactions were assayed by biotinylated oligonucleotide pull down assay. A 0.05 mM double-stranded oligonucleotide that corresponds to parts of the N-cadherin promoter was generated by annealing oligonucleotides (indicated in Table 3) in 500 mM NaCl, 20 mM Tris-HCl (pH 7.5) and 5 mM EDTA. The consensus binding sites for SOX4 are in boldface. 6 µL of dsOligos were coupled with 20 µL of 50% magnetic streptavidin beads slurry (Promega, Madison, USA) in PBS containing 10% of fetal bovine serum for 1 h at room temperature. Eight µg of nuclear extract were used per reaction and added to the previous mixture in 10 mM HEPES, 10 mM KCl, 0.1 mM EDTA, 100 mM NaCl, 2 mM DTT, 1% NP-40 and 1% protease inhibitors for 2 h at 4°C. Beads were washed in PBS containing 1% of Halt Protease Inhibitor Cocktail (Thermo Scientific) and boil in 1× sample buffer. Samples were analyzed by western blotting and probed with anti-Flag antibody (Sigma-Aldrich, Missouri, USA; A8592-1MG: 1∶5000).

**Table 3:** qRT-PCR primer sequences used in the biotinylated oligonucleotide pull down assay.

Gene	Forward primer	Reverse Primer
**N-Cad +29600 Mut**	5' cttgtacaaacaaccccggtatttccaagtgcttacaat 3'	5' attgtaagcacttggaaataccggggttgtttgtacaag 3'
**N-Cad +29600 Wt**	5' cttgtacaaacaacccctttgtttccaagtgcttacaat 3'	5' attgtaagcacttggaaacaaaggggttgtttgtacaag 3'
**N-Cad +25000 Mut**	5' tgcctggggaataaaaaggagttcagtgtcgccgg 3'	5' ccggcgacactgaactcctttttattccccaggca 3'
**N-cad +25000 Wt**	5' tgcctggggaataacaatgagttcagtgtcgccgg 3'	5' ccggcgacactgaactcattgttattccccaggca 3'
**N-Cad −1000 Mut**	5' agcggcgcggggaaaacagggacccggcgccgccc 3'	5' gggcggcgccgggtccctgttttccccgcgccgct 3'
**N-Cad −1000 Wt**	5' agcggcgcggggaacaaagggacccggcgccgccc 3'	5' gggcggcgccgggtccctttgttccccgcgccgct 3'
**N-cad −2600 Mut**	5' aaatcatgctgttggagaatctatgcatccatttgatgttaatg 3'	5' cattaacatcaaatggatgcatagattctccaacagcatgattt 3'
**N-cad −2600 Wt**	5' aaatcatgctgttggagactttgtgcatccatttgatgttaatg 3'	5' cattaacatcaaatggatgcacaaagtctccaacagcatgattt 3'
**N-cad −3900 Mut**	5' tactatttttctcaagttggttattcttcaaagtatgtgtga 3'	5' tcacacatactttgaagaataaccaacttgagaaaaatagta 3'
**N-cad −3900 Wt**	5' tactatttttctcaagttttttgttcttcaaagtatgtgtga 3'	5' tcacacatactttgaagaacaaaaaacttgagaaaaatagta 3'

### Luciferase Assays

HMLE or HEK293T cells were grown to 30% confluence in twenty-four wells-plate (Nunc, Roskilde, Denmark) and transfected with a mixture of 0.3 µg DNA and 1.5 µL PEI overnight either co-transfected with Sox4-reporter luciferase construct or *CDH2*-promoter luciferase construct. After 48 hours of transfection, cells were washed twice with PBS and lysed in 50 µL of passive lysis buffer (Promega, Leiden, The Netherlands) for 20 minutes. 20 µL of the cell lysate was assayed for luciferase activity using Dual–Luciferase Reporter Assay System (Promega) as well as for protein expression analysis by western blotting using anti-Flag antibody (Sigma-Aldrich, Missouri, USA; A8592-1MG: 1∶5000).

## Results

### Identification of SOX4 as a TGF-β-induced Transcription Factor during EMT

To identify novel transcriptional activators potentially regulated by TGF-β we analyzed publicly available gene-expression datasets [9]. These datasets comprise genome-wide expression data from HMLE cells treated with TGF-β for 12 days and the corresponding untreated controls. Differential gene expression analysis focusing on significantly regulated genes increased over 2-fold, and Gene-Ontology analysis using DAVID, revealed the TGF-β-induced expression of several genes belonging to the “DNA-dependent, positive regulation of transcription” GO-term (Table 4). This group of genes included three transcriptional activators *PBX1, SOX4* and *ETS2*, which have been linked to breast cancer tumorigenesis [10], [11], [12]. SOX4 is of particular interest since reduced expression through either the endogenous miR-335 or shRNA-mediated knockdown severely impairs the metastatic capacity of MDA-MB-231 cells in mouse xenograft models [11]. Therefore, we further explored the role of *SOX4* downstream of TGF-β in HMLEs.

**Table 4:** Gene Ontology category ''positive regulation of transcription, DNA-dependent (GO:0045893) genes significantly regulated and greater than 2 fold upregulated during TGF-β-induced EMT.

Gene Name	Fold Change	P value
**zinc finger E-box binding homeobox 1**	11.34	7E-06
**pre-B-cell leukemia homeobox 1**	4.79	0.00303
**transforming growth factor beta 1 induced transcript 1**	2.58	0.0054
**nuclear receptor interacting protein 1**	2.48	0.00501
**nuclear receptor coactivator 1**	2.21	0.00285
**SRY (sex determining region Y)-box 4**	2.19	0.02315
**v-ets erythroblastosis virus E26 oncogene homolog 2 (avian)**	2.11	0.00055

Transcription factors are indicated in bold.

**Figure 1:**
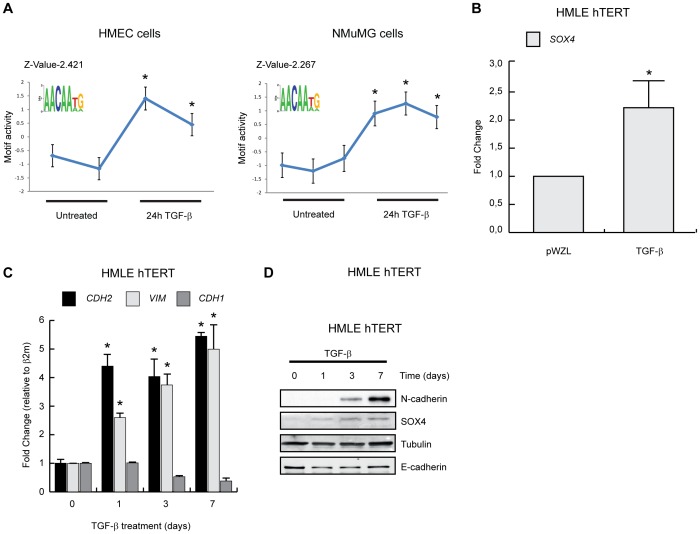
SOX4 expression is increased by TGF-β during EMT. (**A**) MARA analysis predicts Sox activity during EMT in HMEC and NMuMG cells (see text for details) (**B**) Public microarrays databases generated in non-transformed HMEC cells treated with TGF-β or left untreated were analyzed and SOX4 expression was assessed. (**C**) HMLE cells were stimulated with 2.5 ng/mL of TGF-β as indicated, lysed and mRNA expression of *CDH2* (N-cadherin), *VIM* (vimentin), *CDH1* (E-cadherin) were analyzed by qRT-PCR. (**D**) HMLE cells were stimulated with 2.5 ng/mL of TGF-β as indicated and lysed. The protein lysates were visualized by Western-Blotting using anti-N-cadherin, anti-SOX4, anti-Tubulin and anti-E-cadherin antibodies. Western blot data is representative of at least three independent experiments. *p<0,05 (N = 3±SD).

To determine whether TGF-β treatment of mammary epithelial cells and associated increased expression of SOX4 is accompanied by elevated SOX transcriptional output, we performed Motif Activity Response Analysis (MARA). This interrogates transcription factor DNA binding site motifs to determine the transcription factors driving gene expression changes [13]. We used MARA to analyse two independent publicly available datasets of TGF-β treated mouse and human mammary epithelial cells (GSE13986 and GSE28448) [14], [15]. Despite the lack of a specific SOX4 binding motif present in the software, MARA analysis of both HMLE-Tert/Ras cells and normal murine mammary gland (NMuMG) cells treated with TGF-β for 24 h revealed a significant increase in the regulation of genes possessing a SOX binding motif, as exemplified by SOX2 (Fig. 1A). This suggests an increase in the transcriptional output of TGF-β regulated SOX transcription factors. TGF-β treatment resulted in increased *SOX4* expression by over two-fold in the microarray datasets previously analyzed (Fig. 1B).

To confirm TGF-β-mediated regulation of *SOX4* during EMT, HMLE cells were stimulated with TGF-β for 7 days and both protein and mRNA samples were harvested at the indicated time points. Quantitative real-time PCR analysis demonstrated that TGF-β potently induced EMT in HMLE cells as illustrated by the increased expression of *CDH2* (N-cadherin) and *VIM* (vimentin) and a decrease in *CDH1* (E-cadherin) expression (Fig. 1C). SOX4 mRNA expression was also transiently increased upon TGF-β treatment of HMLE cells (Fig. S1A). Western blot analysis of cell lysates obtained from identically treated HMLE cells demonstrated that SOX4 protein expression was also induced by TGF-β in a time dependent manner (Fig. 1D).

Taken together these data indicate that SOX4 expression is regulated by TGF-β in mammary epithelial cells, which correlates with differential expression of genes containing a SOX-motif in their upstream promoters. This suggests that the TGF-β-induced SOX4 transcriptional response may play a role in the process of EMT.

### Conditional Activation of Sox4 is Sufficient to Drive Expression of Mesenchymal Markers

**Figure 2:**
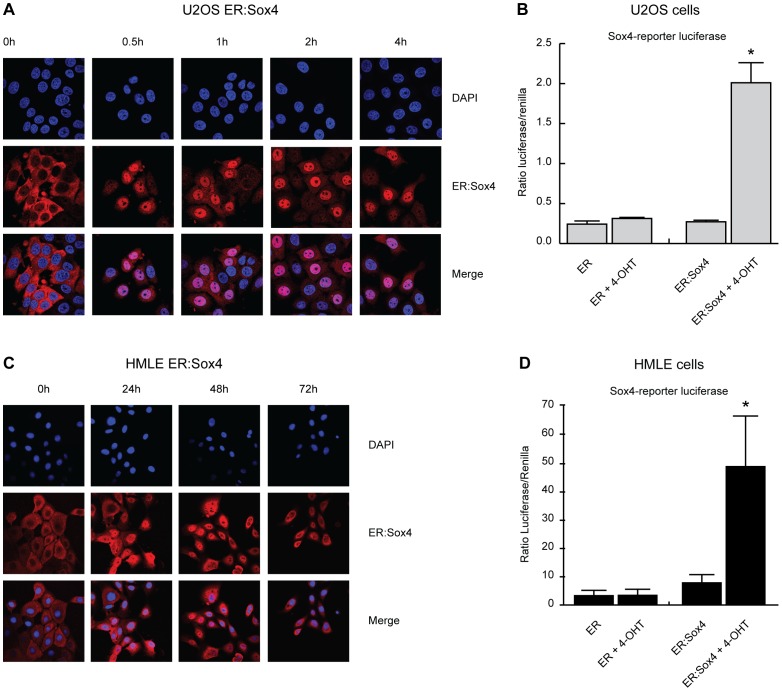
Generation of a Sox4 conditional activation system. The hormone binding domain of the ER was fused to the N-terminus of Sox4. (**A**) ER:*Sox4* was stably transduced in U2OS cells. Immuno-fluorescence analysis of ER:Sox4 localization using an anti-ER antibody after stimulation with 4-OHT (100 nM) for the time points indicated. (**B**) U2OS cells expressing ER and ER:Sox4 were transfected with an optimal Sox4 luciferase reporter construct and treated overnight with 4-OHT (100 nM) after which luciferase activity was measured. (**C**) ER:Sox4 localization in HMLE cells in presence and absence of 4-OHT. (**D**) HMLE cells expressing ER and ER:Sox4 were transfected with an optimal Sox4 luciferase reporter construct and stimulated overnight with 4-OHT (100 nM) after which luciferase activity was measured. Confocal microscopy data is representative of at least three independent experiments. *p<0,05 (N = 3±SD).

To determine whether SOX4 activation is sufficient to induce EMT, we generated a conditional activation system to control Sox4 activation in transduced HMLE cells. Conditional activation of Sox4 was obtained through N-terminal fusion of Sox4 with the estrogen receptor (ER) hormone binding domain generating an ER:Sox4 fusion protein (see Materials and Methods). Through a mutation in the ligand binding domain the ER is no longer responsive to estrogen but is exquisitely sensitive to the synthetic ligand 4-hydroxy-tamoxifen (4-OHT) [16]. In the absence of its ligand, ER is retained in the cytoplasm through association with heatshock and chaperone proteins where it is rapidly degraded. Upon binding of the ligand these proteins dissociate and allow translocation of ER into the nucleus, a property which is conferred to the chimeric ER:Sox4 transcription factor. To initially validate conditional activation of the ER:Sox4 fusion protein, U2OS cells expressing the construct were analyzed for the subcellular localization of ER:Sox4. Cells were treated with 100 nM of 4-OHT and subsequently fixed and permeabilized. Localization of the construct was analyzed using an anti-ERα antibody. As expected ER:Sox4 was exclusively present in the cytoplasm, but rapidly translocated to the nucleus upon stimulation with 4-OHT, where it is retained for the duration of the stimulus (Fig. 2A). To investigate the effect of 4-OHT on Sox4 transcriptional output, luciferase assays were performed using a luciferase reporter construct containing a Sox4 responsive promoter [17]. U2OS cells expressing ER or ER:Sox4 were transfected with the Sox4-reporter luciferase construct and subsequently stimulated overnight with 4-OHT. Addition of 4-OHT resulted in a strong induction of the luciferase reporter, whereas no activity was observed in the absence of 4-OHT or in the treated and untreated control cell lines stably transduced with merely the ER-hormone binding domain (Fig. 2B). The ER:Sox4 fusion construct thus allows for conditional and robust activation of Sox4 by 4-OHT.

**Figure 3:**
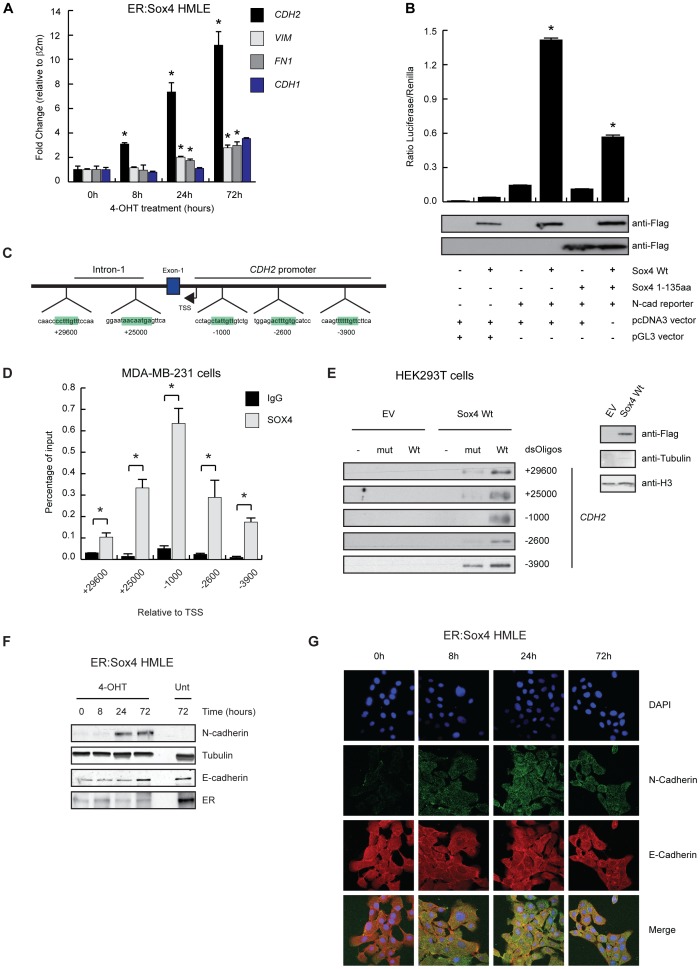
Sox4 activation induces upregulation of mesenchymal markers. (**A**) HMLE cell lines expressing ER:Sox4 were stimulated with 4-OHT (100 mM) as indicated. Cells were lysed and mRNA expression of *CDH2* (N-cadherin), *VIM* (vimentin), *FN1* (fibronectin) and *CDH1* (E-cadherin) was analyzed by qRT-PCR. (**B**) HEK293T cells were transiently transfected with Flag-tagged Sox4 Wt or Flag-tagged Sox4 1-135aa and co-transfected with a *CDH2* luciferase reporter construct as indicated. After 48 hours luciferase activity was measured. Protein expression was assayed by Western blotting using anti-Flag antibody. (**C**) Schematic representation of the *CDH2* promoter region and predicted Sox4 binding sites. (**D**) Chromatin Immunoprecipitation (ChIP) assay using IgG and SOX4 antibodies in MDA-MB-231 cells. Real time PCR was performed using *CDH2* promoter-specific primers to test SOX4 occupancy at this region. (**E**) HEK293T cells were transiently transfected with the empty vector pcDNA3 or Flag-tagged Sox4 Wt. After 48 hours cells were harvested and nuclear fraction was extracted. Nuclear extracts were used to perform a biotinylated oligonucleotide pull down assay in which three *CDH2* promoter sites and two sites localized in the first intron of *CDH2* were included. Lysates were assessed by western blotting using anti-Flag antibody. (**F**) HMLE cell lines expressing ER:Sox4 were stimulated with 4-OHT (100 nM) as indicated or left untreated. Cells were lysed and lysates were analyzed by Western blotting using anti-N-cadherin, anti-Tubulin, anti-E-cadherin and anti-ER antibodies. (**G**) HMLE cells expressing ER:Sox4 were treated with 4-OHT (100 nM) as indicated. Cells were fixed, permeabilized and the expression of N-cadherin and E-cadherin was assessed (green and red respectively). Blue = DAPI. Western blot and confocal microscopy data is representative of at least three independent experiments. *p<0,05 (N = 3±SD).

We subsequently used this system to test whether SOX4 induced gene expression changes could contribute to EMT. To this end we generated polyclonal HMLE lines expressing ER:Sox4. Similar to U2OS cells, ER:Sox4 localization in HMLE cells was dependent on 4-OHT resulting in sustained translocation from the cytoplasm to the nucleus, and robustly activated the Sox4 responsive promoter in luciferase assays (Fig. 2C and Fig. 2D). Subsequently, ER:Sox4 HMLE cells were treated with 4-OHT as indicated after which mRNA was isolated and analyzed by qRT-PCR for expression changes in both epithelial and mesenchymal markers. Sox4 activation alone was sufficient to induce expression of mesenchymal markers including *CDH2* (N-cadherin), *VIM* (vimentin) and *FN1* (fibronectin), whereas expression of *CDH1* (E-cadherin) remained unaltered (Fig. 3A). No expression changes in epithelial and mesenchymal markers were observed in the ER HMLE cells (Fig. S2A). Since the SOX-family member SOX9 has been demonstrated to directly regulate N-cadherin expression in chondrocytic CFK2 cells, we investigated SOX4 mediated activation of the *CDH2* promoter in more detail [18]. In order to test SOX4 mediated activation of the *CDH2* gene we performed luciferase assays in HEK293 cells using a *CDH2*-promoter luciferase construct (kindly provided by Dr. David Goltzman). Co-transfection of Sox4 and the *CDH2*-promoter luciferase construct resulted in a potent induction of luciferase expression compared to control transfected cells. Only a minor effect of Sox4 was observed on the control pGL3 reporter lacking the *CDH2*-promoter (Fig. 3B). Moreover, Sox4 mediated activation of the *CDH2*-promoter could be inhibited by overexpression of a dominant negative Sox4 construct (Sox4 1-135aa), consisting of the N-terminal region and DNA-binding domain thereby blocking Sox4 DNA-binding (Fig. 3B) [19]. These findings indicate that Sox4 expression is sufficient to induce *CDH2* expression and most likely depends on its DNA-binding. Next, we wished to determine whether SOX4 could bind to the *CDH2* promoter [18]. Bioinformatic analysis using the ContraV2 software revealed several highly conserved SOX4 binding motifs in the promoter region and the first intron of the *CDH2* gene (Fig. 3C) [20]. We subsequently analyzed SOX4 binding to these conserved motifs using chromatin immuno-precipitation followed by qRT-PCR (ChIP-qPCR) in metastatic MDA-MB-231 breast cancer cells express high levels of mesenchymal markers. The SOX4 ChIP showed a significant degree of enrichment for five of the conserved binding sites compared to the IgG control, indicating that SOX4 can bind the *CDH2* promoter on these sites (Fig. 3D). In order to confirm SOX4 binding to these sites we performed biotin-labeled oligonucleotide pull down assays using the identified SOX4 binding sites and mutated versions hereof. HEK293 cells were transfected with flag-tagged Sox4 or empty vector and a biotin-labeled oligonucleotide pulldown was performed on the nuclear lysates. Western blot analysis revealed binding to all the *CDH2* promoter sites, whereas little or no binding was detected in the empty vector control and mutated probes (Fig. 3E). This confirms the potential of SOX4 to bind to these sites in the *CDH2* promoter.

To assess whether changes induced by Sox4 on the *CDH2* and *CDH1* mRNA levels also result in alterations in protein expression we investigated protein expression of N-cadherin and E-cadherin. ER:Sox4 HMLE cells were treated with 4-OHT and E-cadherin and N-cadherin expression were analyzed. In accordance with qRT-PCR results, Sox4 activation induced expression of N-cadherin whereas E-cadherin expression was not down-regulated (Fig. 3E). No changes in N-cadherin or E-cadherin expression were observed in ER HMLE cells (Fig. S2B). Next, N-cadherin and E-cadherin expression and localization was analyzed by immuno-fluorescence microscopy after activation of Sox4 for the indicated time-points. Sox4 activation again resulted in increased expression of N-cadherin without affecting the levels of E-cadherin expression (Fig. 3G). Since TGF-β-mediated downregulation of *CDH1* may only occurs after three days [21], we investigated whether prolonged activation of Sox4 by 4-OHT for four days could result in reduced *CDH1* expression. As expected, qRT-PCR analysis revealed that continued Sox4 activation increased expression of the mesenchymal markers *CDH2*, *VIM* and *FN1*, but did not result in downregulation of the epithelial markers *CDH1* and *CTNNB1* (β-Catenin) (Fig. S2C, left panel). No changes were observed in the ER HMLE cells (Fig. S2C, right panel). Consistent with the qRT-PCR results, western blot analysis demonstrated that prolonged 4-OHT treatment of ER:Sox4 HMLE cells resulted in upregulation of N-cadherin, but did not result in altered expression of the epithelial markers E-cadherin and β-Catenin (Fig. S2D). No alterations in the expression of epithelial and mesenchymal markers was observed in the ER HMLE cells (Fig. S2D). In addition, immuno-fluorescence microscopy showed no change in E-cadherin expression in both the ER:SOX4 and ER HMLE cells after 4 day stimulation with 4-OHT (Fig. S2E) Thus, Sox4 activation is sufficient to induce expression of the mesenchymal marker N-cadherin in HMLE cells without altering E-cadherin expression.

### SOX4 Knockdown Delays TGF-β-induced Expression of Mesenchymal Markers during EMT

**Figure 4:**
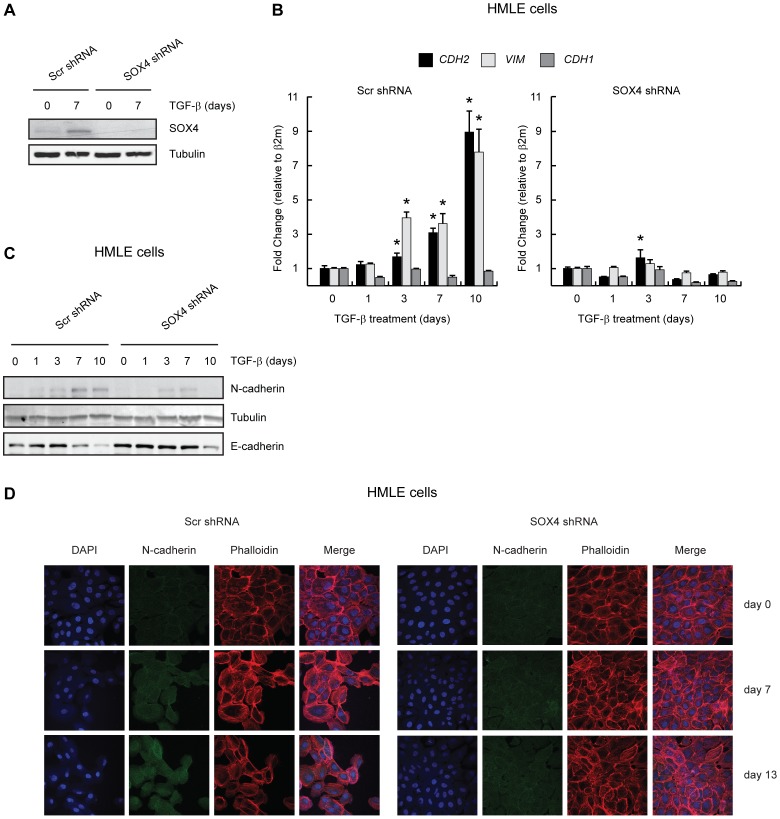
SOX4 knockdown delays TGF-β-induced EMT. HMLE cells line were either transduced with a control shRNA (Scr shRNA) or with a shRNA targeting SOX4 (SOX4 shRNA). (**A**) Scr shRNA and SOX4 shRNA cell lines were either treated with 2.5 ng/mL of TGF-β for 7 days or left untreated. Cells were lysed and analyzed by Western Blotting using anti-SOX4 and anti-Tubulin antibodies (**B**) HMLE cell lines expressing Scr shRNA and SOX4 shRNA were stimulated with 2.5 ng/mL of TGF-β as indicated. Cells were lysed and mRNA expression of *CDH2* (N-cadherin), *VIM* (vimentin) and *CDH1* (E-cadherin) were assessed. (**C**) Cell lysates of HMLE cell lines expressing Scr shRNA and SOX4 shRNA stimulated with 2.5 ng/mL of TGF-β as indicated and analyzed by western blotting using N-cadherin, anti-Tubulin and anti-E-cadherin antibodies. (**D**) Scr shRNA and SOX4 shRNA cell lines were stimulated with 2.5 ng/mL of TGF-β as indicated. Cells were fixed, permeabilized and N-cadherin expression was assessed (green). Blue = DAPI; red = phallodin. Western blot and confocal microscopy data is representative of at least three independent experiments. *p<0,05 (N = 3±SD).

Since we observed that SOX4 can induce expression of N-cadherin, we examined whether the TGF-β-mediated induction of N-cadherin is dependent on SOX4 expression. To this end, SOX4 knockdown was performed in HMLE cells using lentiviral shRNA constructs. Western blot analysis of SOX4 expression showed efficient depletion of SOX4 in both the presence and absence of TGF-β, whereas this not affected in the scrambled control HMLE cells (Fig. 4A). SOX4 knockdown was maintained during the course of the experiment, as assed by Western blot analysis on day 7 (Fig. 4A) To assess whether SOX4 knockdown affects TGF-β-mediated regulation of N-cadherin and vimentin, scrambled and SOX4 shRNA HMLE cells were treated with TGF-β for 10 days and mRNA and protein isolated at the indicated time points. *CDH2* and *VIM* mRNA expression, as determined by qRT-PCR, was effectively induced upon TGF-β treatment in the scrambled HMLE cells (Fig. 4B, left panel). In contrast, in the SOX4 knockdown HMLE cells TGF-β-mediated induction of *CDH2* and *VIM* was strongly reduced (Fig. 4B, right panel). Furthermore, Western blot analysis revealed that on the protein level SOX4 knockdown also reduces the TGF-β-mediated induction of N-cadherin (Fig. 4C). Similarly, immuno-fluorescence microscopy demonstrated that after 10 days of TGF-β-mediated EMT induction, the SOX4 knockdown HMLE cells expressed less N-cadherin than the scrambled control cells (Fig. 4D). Taken together, these data show that TGF-β-mediated regulation of SOX4 expression and activity is required for efficient induction of N-cadherin and potentially other mesenchymal markers.

## Discussion

Tumor metastasis is the major cause of cancer-related death and in a wide-variety of tumors the epithelial-to-mesenchymal transition has been demonstrated to contribute to this process. EMT is characterized by the loss of epithelial makers and acquisition of mesenchymal traits, confers invasive, chemotherapy resistance and stem cell properties to tumor cell [4], [22]. Cancers exploit this developmental process and as a result can acquire a more invasive and metastatic phenotype. The TGF-β signaling pathway is one of the most potent and well-studied inducers of EMT during both embryonic development and cancer progression. Here, we demonstrate that expression of the developmental transcription factor SOX4 increases during TGF-β-induced EMT and can contribute to the change in cellular phenotype by controlling the expression of mesenchymal markers in human mammary epithelial cells.

In the developing mouse SOX4 is highly expressed in mesenchymal tissues and at variable levels in other tissues [23], [24], [25]. In combination with SOX11 and SOX12, two additional members of the SOXC group of transcription factors, SOX4 has been demonstrated to be vital for the survival of neuronal and mesenchymal progenitor cells, indicating that also during development SOX4 contributes to acquisition of neuronal and mesenchymal properties [26]. Accordingly, SOX4 plays an important role during embryonic development and SOX4 knockout mice die at embryonic day 14.5 due to defective formation of the heart, but SOXC knockouts also suffer from additional defects such as a cleft lip caused by defective palate fusion [24], [27]. Interestingly, during embryonic development TGF-β-induced EMT is particularly prominent in both the formation of the heart and palatal fusion, potentially suggesting that defective TGF-β-induced EMT contributes to the SOX4 knockout cardiac and palate phenotype [22], [28].

Despite the prominent role during embryonic development very little is known about the regulation of SOX4 on the post-translational level. We have recently shown that SOX4 is rapidly degraded and can be stabilized through its interaction with the adaptor protein syntenin [19]. Interestingly, syntenin has been demonstrated to be regulated by in a number of signal transduction pathways including the WNT, IL-5, TGFα and Syndecan-regulated signaling pathways, suggesting that Syntenin-mediated regulation of SOX4 protein stability and activity could be involved in embryonic and tumorigenic processes mediated by these signaling events [17], [19], [29], [30].

Similar to TGF-β, SOX4 has a paradoxical function in tumorigenesis potentially acting as both a tumor-suppressor and promoter of tumor progression [25]. High levels of SOX4 mRNA expression are present in nearly all major human cancers and SOX4 has been recognized as one of the 64 cancer signature genes [31]. Despite its elevated expression in human cancers, the transcriptional changes mediated by SOX4 remain poorly defined. A number of studies have investigated SOX4 mediated transcriptional changes in the context of prostate, hepatocellular carcinoma (HCC), small cell lung cancer and adenoid cystic carcinoma, resulting in the identification of a large number of potential SOX4 targets [32], [33], [34], [35]. However, it remains to be determined whether most of the identified genes are indeed direct transcriptional targets of SOX4 or are the result of secondary events. Additionally, there is very little overlap in the transcriptional targets identified in different tumors, suggesting that, similar to TGF-β, the transcriptional response initiated by SOX4 is highly context dependent.

SOX4 has also been demonstrated to contribute to cancer progression and metastasis in breast cancer glioma and HCC. Similar to breast cancer, HCC metastasis can be driven by TGF-β through EMT induced phenotypic changes [36]. In HCC, SOX4 expression is greatly elevated in metastatic tumors compared to their non-metastatic counterparts, and shRNA-mediated SOX4 knockdown in metastatic HCC cells significantly reduced tumor metastasis [35]. In addition to the reduced metastatic capacity, SOX4 knockdown HCC cells were observed to have alterations in cell morphology and showed decreased expression of the mesenchymal markers vimentin, suggesting that shRNA-mediated reduction in the expression of SOX4 in metastatic HCC cells reverts their mesenchymal phenotype to an epithelial phenotype through a mesenchymal to epithelial transition (MET) [35]. *SOX4* has been demonstrated to be a downstream target of the TGF-β signaling pathway in a number of cell types including T-helper cells and glioma [37], [38]. In glioma, SOX4 expression is directly induced by TGF-β activated SMAD2/3, resulting in the maintenance of sternness and tumorigenicity [37]. Interestingly, SOX4 expression is also increased in normal mammary stem cells, and together with other mammary stem cells markers identifies the cancer stem cell content of breast cancers [39]. Similarly, induction of EMT in breast cancers generates increased stem cell content and confers stem cell properties [3]. It is thus possible that similar to glioma, TGF-β-induced breast cancer stem cells properties are mediated by SOX4, suggesting that SOX4 induction might impact on multiple aspects of the EMT phenotype. Indeed, a recent study has shown that SOX4 promotes EMT in immortalized human mammary epithelial cell line MCF10A, which was associated with a mesenchymal phenotype, enhanced stem cell properties, increased cellular migration and invasion *in vitro* and increased RAS induced tumorigenesis *in vivo* [40]. Additionally, SOX4 was demonstrated to be positively regulated by TGF-β and was essential in the TGF-β-mediated induction of EMT. Moreover, in patient breast cancer samples SOX4 expression correlated with tumor-grade and triple negative breast cancers. The SOX4 mediated induction of EMT was linked to increased expression of the EMT-inducing transcription factor ZEB1. However, direct transcriptional regulation was not determined and examination of the *ZEB1* promoter revealed no SOX4 binding sites, suggesting that SOX4 mediated regulation of ZEB1 could be indirect.

Taken together, these findings suggest that SOX4 could mediate TGF-β-induced effects, such as EMT and maintenance of cancer stem cells in a variety of tumors, thereby contributing to tumor metastasis and progression. It is also possible that the tumor-suppressive roles of SOX4 mirror the effect TGF-β in these cell types, indicating that similar to TGF-β the outcome of SOX4 activation might be highly dependent on tumor stage and signals provided by the tumor microenvironment.

TGF-β-mediated induction of SOX4 and subsequent increased expression of N-cadherin could be sufficient to drive tumor metastasis even in the absence of a concomitant decrease in E-cadherin expression. Ectopic expression of N-cadherin in epithelial breast cancer cell lines has been demonstrated to be sufficient to promote migration and invasion, regardless of continued E-cadherin expression [41]. In addition, in a transgenic mouse model, mammary epithelial specific coexpression of polyomavirus middle T antigen (PyVmT) and N-cadherin potentiated pulmonary metastasis *in vivo* and increased motility and invasion *in vitro* compared to control PyVmT mice, in the presence of comparable E-cadherin expression [6]. Moreover, it has recently been described that, in a mouse model of pancreatic cancer, N-cadherin haploinsufficiency increases survival [42]. It thus appears that the metastasis promoting activity of N-cadherin dominates over the suppressive function of E-cadherin, suggesting that a complete transition might not be required for the induction of a metastatic phenotype in breast cancer cells. In addition, ectopic expression of N-cadherin in a number of prostate cancer cell lines was demonstrated to be sufficient to induce invasion and metastasis and was able to confer an EMT associated phenotype as illustrated by loss of E-cadherin, mesenchymal morphology and increased expression of vimentin [43]. This suggests that continued expression of N-cadherin is sufficient for the increased expression of additional mesenchymal markers and EMT in these cells. Thus, in transformed cells forced expression of SOX4 and the associated increase in N-cadherin expression could be sufficient to drive EMT.

Identification of the molecular mechanisms underlying the development of EMT is imperative to improve our understanding of tumorigenesis and will aid in the development of future cancer therapeutics targeting the development of cancer metastasis. The role of SOX4 in this processes warrants further investigation into its function and regulation. Future insight into the regulation of SOX4 and its downstream target genes in the context of cancer development and progression, could prove useful to design pharmacological compounds which modulate the activity of this important transcription factor.

## Supporting Information

Figure S1 **SOX4 mRNA expression increases upon TGF-β stimulation.** **(A)** HMLE cells were stimulated with 2.5 ng/mL of TGF-β as indicated, lysed and mRNA expression of SOX4 was analysed by qRT-PCR. *p<0,05 (N = 3±SD).
(TIF)

Figure S2 **Sox4 activation is insufficient to down regulate epithelial markers.** **(A)** HMLE cell lines ER were stimulated with 4-OHT (100 mM) as indicated. Cells were lysed and mRNA expression of *CDH2* (N-cadherin), *VIM* (vimentin), *FN1* (fibronectin) and *CDH1* (E-cadherin) was analyzed by qRT-PCR. **(B)** HMLE cell lines expressing ER:Sox4 and ER were stimulated with 4-OHT (100 nM) as indicated or left untreated. Cells were lysed and lysates were analyzed by Western blotting using anti-N-cadherin, anti-Tubulin, anti-E-cadherin and anti-ER antibodies. **(C)** HMLE cell lines expressing ER:Sox4 or ER were stimulated with 4-OHT (100 nM) for 96 hours or left untreated. Cells were lysed and mRNA expression of *CDH2* (N-cadherin), *VIM* (vimentin), *FN1* (fibronectin), *CDH1* (E-cadherin) and *CTNNB1* (β-catenin) was analyzed by qRT-PCR. In addition **(D)** Protein expression of N-cadherin, Sox4, β-catenin, E-cadherin and tubulin was assessed by western bloting using the respective antibodies. **(E)** HMLE cell lines expressing ER:Sox4 or ER were stimulated with 4-OHT (100 nM) as indicated. Cells were fixed, permeabilized and the expression of N-cadherin and E-cadherin was visualized by confocal microscopy (green and red respectively). Blue = DAPI. Western blot and confocal microscopy data is representative of at least three independent experiments. *p<0,05 (N = 3±SD).
(TIF)
