# Supplementary figures and images for: SOX4 Mediates TGF-β-Induced Expression of Mesenchymal Markers during Mammary Cell Epithelial to Mesenchymal Transition
Source: PLoS One. 2013 Jan 3;8(1):e53238. doi: 10.1371/journal.pone.0053238 (PMC3536747; doi:10.1371/journal.pone.0053238)

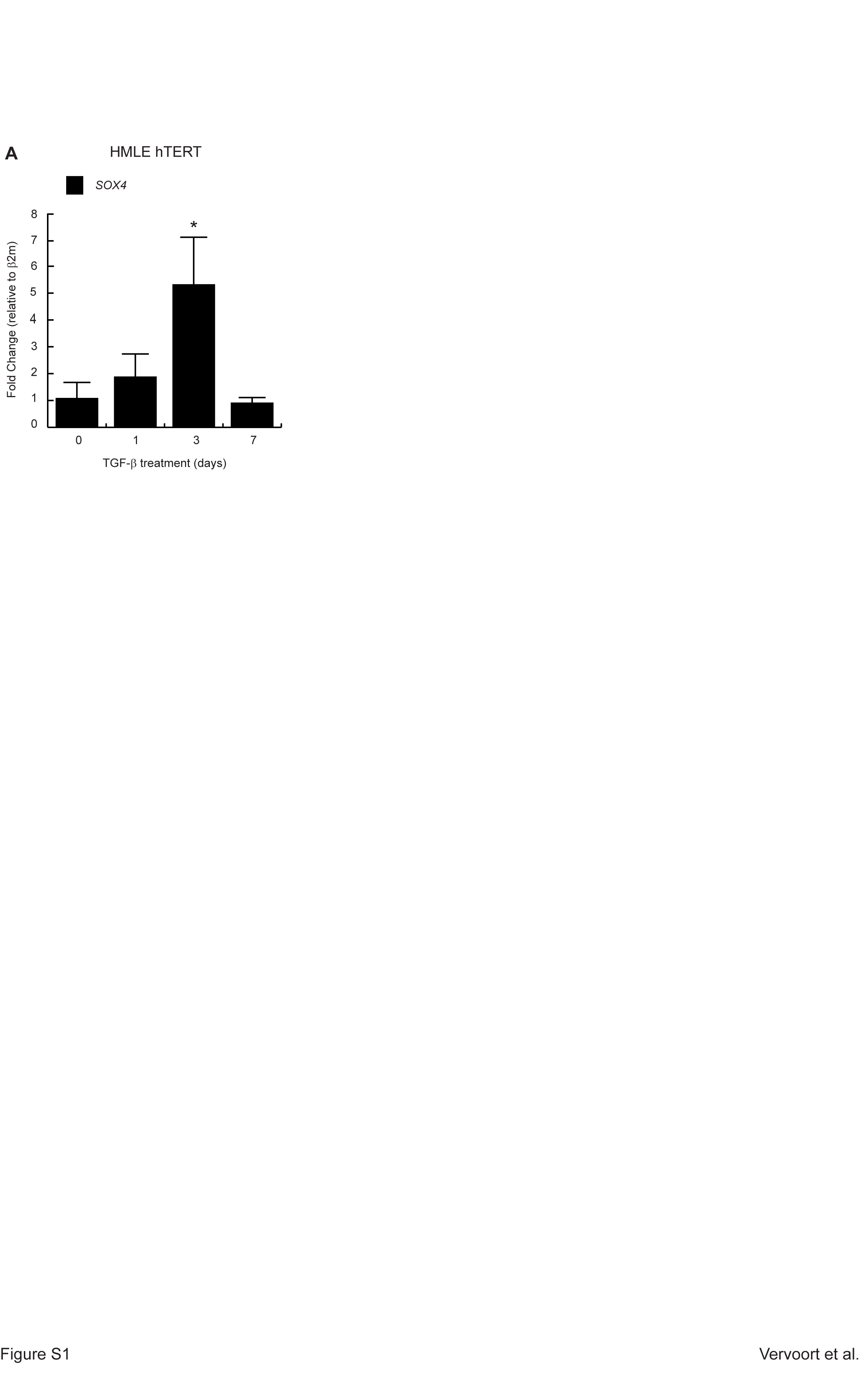

Supplement: Figure S1 — SOX4 mRNA expression increases upon TGF-β stimulation. (A) HMLE cells were stimulated with 2.5 ng/mL of TGF-β as indicated, lysed and mRNA expression of SOX4 was analysed by qRT-PCR. *p<0,05 (N = 3±SD). (TIF) [file pone.0053238.s001.tif]

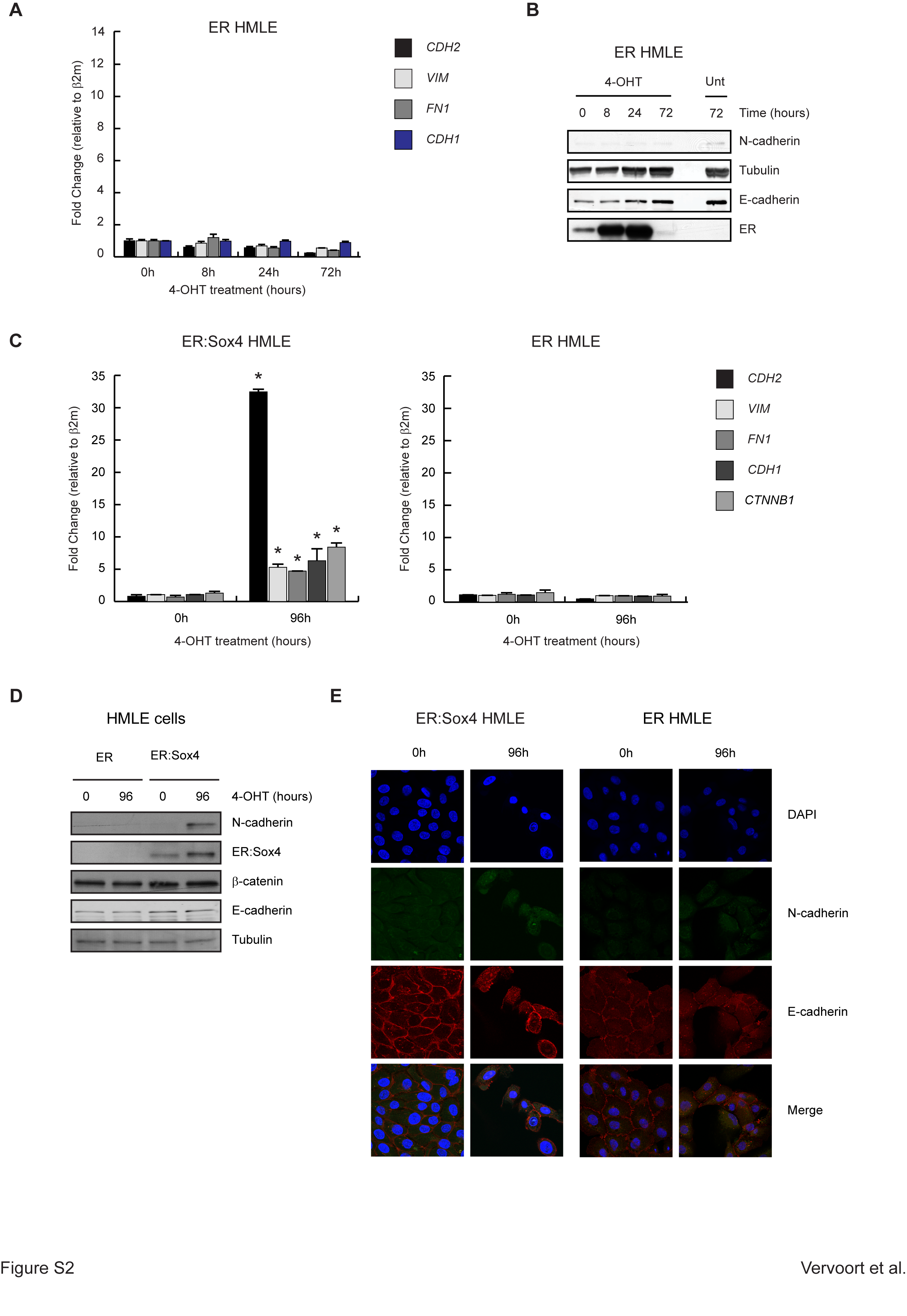

Supplement: Figure S2 — Sox4 activation is insufficient to down regulate epithelial markers. (A) HMLE cell lines ER were stimulated with 4-OHT (100 mM) as indicated. Cells were lysed and mRNA expression of CDH2 (N-cadherin), VIM (vimentin), FN1 (fibronectin) and CDH1 (E-cadherin) was analyzed by qRT-PCR. (B) HMLE cell lines expressing ER:Sox4 and ER were stimulated with 4-OHT (100 nM) as indicated or left untreated. Cells were lysed and lysates were analyzed by Western blotting using anti-N-cadherin, anti-Tubulin, anti-E-cadherin and anti-ER antibodies. (C) HMLE cell lines expressing ER:Sox4 or ER were stimulated with 4-OHT (100 nM) for 96 hours or left untreated. Cells were lysed and mRNA expression of CDH2 (N-cadherin), VIM (vimentin), FN1 (fibronectin), CDH1 (E-cadherin) and CTNNB1 (β-catenin) was analyzed by qRT-PCR. In addition (D) Protein expression of N-cadherin, Sox4, β-catenin, E-cadherin and tubulin was assessed by western bloting using the respective antibodies. (E) HMLE cell lines expressing ER:Sox4 or ER were stimulated with 4-OHT (100 nM) as indicated. Cells were fixed, permeabilized and the expression of N-cadherin and E-cadherin was visualized by confocal microscopy (green and red respectively). Blue = DAPI. Western blot and confocal microscopy data is representative of at least three independent experiments. *p<0,05 (N = 3±SD). (TIF) [file pone.0053238.s002.tif]
